# Psychometric properties of the post-traumatic stress disorder checklist for DSM-5 (PCL-5) in Chinese stroke patients

**DOI:** 10.1186/s12888-022-04493-y

**Published:** 2023-01-09

**Authors:** Che Jiang, Gaici Xue, Shujing Yao, Xiwu Zhang, Wei Chen, Kuihong Cheng, Yibo Zhang, Zhensheng Li, Gang Zhao, Xifu Zheng, Hongmin Bai

**Affiliations:** 1 Department of Neurosurgery, General Hospital of Southern Theatre Command, 111 Liuhua Road, Guangzhou, 510010 China; 2 School of Psychology, South China Normal University, Guangzhou, 510631 China; 3 Department of Neurology, General Hospital of Southern Theatre Command, Guangzhou, 510010 China

**Keywords:** Stroke, PTSD, PCL-5, CAPS-5, Psychometric property

## Abstract

**Background:**

Stroke is a devastating disease and can be sufficiently traumatic to induce post-traumatic stress disorder (PTSD). Post-stroke PTSD is attracting increasing attention, but there was no study assessing the psychometric properties of the PCL-5 in stroke populations. Our study was conducted to examine the psychometric properties of the PTSD Checklist for DSM-5 (PCL-5) in Chinese stroke patients.

**Methods:**

This was a cross-sectional observational study conducted at our hospital. Three hundred and forty-eight Chinese stroke patients came to our hospital for outpatient service were recruited. They were instructed to complete the PCL-5 scales and were interviewed for PTSD diagnosis with the Clinician Administered PTSD Scale for DSM-5 (CAPS-5). The cutoff scores, reliability and validity of the PCL-5 were analyzed.

**Results:**

PCL-5 scores in our sample were positively skewed, suggesting low levels of PTSD symptoms. The reliability of PCL-5 was good. Exploratory and confirmatory factor analyses indicated acceptable construct validity, and confirmed the multi-dimensionality of the PCL-5. By CFA analysis, the seven-factor hybrid model demonstrated the best model fit. The PCL-5 also showed good convergent validity and discriminant validity. Receiver operating characteristic (ROC) analyses revealed a PCL-5 score of 37 achieved optimal sensitivity and specificity for detecting PTSD.

**Conclusions:**

Our findings supported the use of PCL-5 as a psychometrically adequate measure of post-stroke PTSD in the Chinese patients.

## Background

Post-traumatic stress disorder (PTSD) is a psychiatric disorder which develops as a result of exposure to life-threatening traumatic events. According to the Statistical Manual of Mental Disorders-version 5 (DSM-5), PTSD is characterized by four clusters of symptoms: recurrent involuntary intrusive memories, avoidance, negative alterations in cognition and mood, and alterations in arousal and reactivity [1]. The PTSD Checklist (PCL) is one of the most frequently used self-report screener for PTSD, and the PCL-5 is the updated version that conforms to the DSM-5 criteria [2]. Compared to PCL-M/PCL-C/PCL-S which conforms to the DSM-IV criteria, PCL-5 is the only version designed to evaluate the severity of PTSD symptoms across various types of traumas. It has been translated to several languages and showed good internal consistency, test–retest reliability, and validity in different samples [3–6]. The Chinese version of PCL-5 has been translated and used in traumatized samples [7–9].

Most previous studies focused on the PTSD secondary to external traumatic events such as combat and sexual violence, while less studies focused on medical conditions or acute illness [4, 10, 11]. Stroke is a serious health event and presents one of the leading causes of death and disability in the worldwide [12]. It has subtypes like cerebral infarction, intracerebral hemorrhage, transient ischemic attack (TIA), and subarachnoid hemorrhage (SAH). According to a meta-analysis on post-stroke mood disorder, the prevalence of major depressive disorder, anxiety, dysthymia, and adjustment disorder were 17.7%, 9.8%, 3.1%, and 6.9% respectively [13]. Stroke features sudden onset of neurologic deficits and potentially threatens patients’ lives, which kind of medical events is qualified as traumatic events according to the DSM-5 criteria. Due to sample heterogeneity, the prevalence of PTSD (or PTSD symptom) varies greatly, ranging from 4 to 37% [14]. Although some studies showed that the degree of disability caused by stroke correlates positively with PTSD symptoms [15–18], mild stroke [19] and TIA [20] can also lead to PTSD.

China bears the biggest stroke burden in the world, and stroke is associated with the highest disability-adjusted life-years lost in this country [21]. Annual estimates of 11 million prevalent cases and 2.4 million new cases were reported [22]. Due to the recurrent nature of stroke, long-term medical management is especially important. The comorbidity of PTSD is related to stroke patients’ non-adherence of treatment [23, 24], as well as lower quality of life [25, 26]. So, regular psychological assessments using PCL-5 at follow-ups for stroke patients are helpful and deserves wider application. Presently, there are no studies focusing on the psychometric properties of PCL-5 in stroke patients, thus our study aimed at solving this problem in the Chinese sample. Specifically, the evaluation goals were to explore the reliability of the PCL-5, the factor loadings and construct validity by factor analyses, and the convergent and discriminant validity through correlations between the PCL-5 and another measure of PTSD (i.e., CAPS-5) and between the PCL-5 and measures of other constructs (e.g., anxiety, depression, and alcohol abuse), respectively.

## Methods

### Participants

All methods were carried out in accordance with relevant guidelines and regulations. The study was approved by the Ethics Committee of General Hospital of Southern Theatre Command. Chinese stroke patients came to our hospital (PLA General Hospital of Southern Theatre Command) for outpatient service from August 2021 to August 2022 were asked to participate in this survey if the doctors regarded the patients can cooperate. The exclusion criteria were as follows: age < 18 years or > 80 years; severe aphasia or cognitive impairment; refused to participate in this survey. There were no financial or other incentives for taking part, except for the doctor’s encouragement. Over 700 patients were initially invited. Finally, three hundred and forty-eight patients were included, but the rest either refused due to various reasons or were ineligible. All of the participants gave their written consent for participation. The mean age of our sample was 55.41, older than that of most samples in other researches on PTSD. The majority of our participants were male, with a high school or lower educational level, and not in employment. Cerebral infarction represented the major subtype of stroke (Table 1).

**Table 1:** Demographic data

	*M*	*SD*
Age	55.41	10.58
NIHSS score	2.69	2.62
	*Count*	*Percent*
Gender (female)	94	27.01%
Marital status		
Married or cohabiting	255	73.28%
Single	85	24.43%
Educational level		
High school or lower	278	79.89%
University or higher	65	18.68%
Employment status		
Employed	126	36.21%
Unemployed/retired	220	63.22%
Stroke type		
CI	269	77.30%
IH	33	9.48%
TIA	39	11.21%
Other	7	2.01%
Leision site		
Cerebrum	209	60.06%
Cerebellum	22	6.32%
Brainstem	39	11.21%
Other/unknown	78	22.41%
Course of stroke		
< 0.5 year	69	19.83%
0.5–1 year	95	27.30%
> 1 year	184	52.87%

Values are expressed as mean ± SD or percentage

*CI* Cerebral infarction, *IH* Intracerebral hemorrhage, *TIA* Transient ischemic attack, *SAH* Subarachnoid hemorrhage

### Procedure

All participants were asked to complete self-reported scales, and their demographic data were obtained. Further explanation of any items in the PCL-5 were not given by specialists even if participants asked. Then, participants were interviewed using CAPS-5 by masters- and doctoral-level clinicians with formal training on the measure. The assigned interviewers took part in CAPS-5 training workshop and were supervised throughout the study. The training and supervision were conducted by the psychologists listed as the fifth and the second to last authors. The participants were instructed to rate their symptoms only in relation to their stroke experience but not to other traumatic events when completing the PCL-5 and CAPS-5. The CAPS-5 was administered by interviewers without knowledge of the PCL-5 scores. Twenty interviews were audio-recorded for the evaluation of inter-rater reliability. After the survey, participants were thanked for their participation.

### Measures

The Chinese versions of all measures used in our study were adapted by a two-stage process of translation and back translation by bilingual researchers based on the original English versions, and they were further verified by senior experts.

#### Hospital Anxiety and Depression scale (HADS) [27]

This scale has two subscales of depression (HADS-D) and anxiety (HADS-A), each containing 7 items. The items are rated from 0 (most of the time) to 3 (absolutely not). Higher scores indicate greater degree of anxiety or depression. A subscale score of ≥ 8 suggests being anxious or depressed, respectively. The Chinese version showed good internal and test–retest reliability [28–30].

#### Alcohol Use Disorder Identification Test (AUDIT)

This scale is used to assess the habits of alcohol consumption and related hazardousness [31]. It has 10 items and its total score ranges from 0 to 40. Higher score indicates worse alcohol related problems. A total score of ≥ 8 indicates hazardous or harmful drinking. Good psychometric properties have been proved for the Chinese translated version [5, 6, 32–34].

#### Post-traumatic stress disorder checklist for DSM-5 (PCL-5)

The PCL-5 is a 20-item self-report scale evaluating DSM-5 PTSD symptoms [35]. Items 1–5 on the PCL-5 correspond to Criterion B, items 6–7 to Criterion C, items 8–14 to Criterion D, and items 15–20 to Criterion E. The 20 item is rated on a 5-point Likert scale (0 = not at all, 1 = a little bit, 2 = moderately, 3 = quite a bit and 4 = extremely) reflecting the severity of traumatic experience. The scores are summed to create a total severity score (range 0–80). This scale has been validated or use in cardiovascular populations [36] and been frequently used in assessing stroke patients [37, 38]. The Chinese version has been validated and widely used [39–41].

#### The clinician-administered PTSD scale for DSM-5 (CAPS-5) [42]

The CAPS-5 is a scale designed for the diagnosis of PTSD, and is used for semi-structured interview. Its items correspond to DSM-5 criteria of PTSD (criterion A to H), and includes 5 items for criterion B, 2 for criterion C, 7 for criterion D, and 6 for criterion E. Each of the items (criterion B to E) is rated on a Likert (0–4) scale from 0 = absent to 4 = extremely, with a total score ranging from 0 to 80. According to the SEV2 rule for CAPS-5 symptom scoring, a severity rating of 2 (moderate) or higher indicates presence of a symptom or impairment [43]. A PTSD diagnosis is made based on the participant satisfying all DSM-5 criteria, fulfilling at least one item each of intrusion (criterion B) and avoidance (criterion C), two items each of negative changes in cognitions and mood (criterion D) and hyperarousal (criterion E), functional impairment (criterion G) and the presence of symptoms for ≥ 1 month (criterion F). Psychometric evaluation of the CAPS-5 demonstrated good internal consistency, strong inter-rater reliability and test–retest reliability [43].

### Statistical analysis

Data were analyzed using SPSS 18.0 and AMOS 22.0. The skewness for PCL-5 items were first psychometrically examined. The reliability of PCL-5 and other measures were measured. Furthermore, item-total correlations and Cronbach’s alpha-if item deleted for the PCL-5 were reported. Exploratory factor analysis (EFA) was conducted to examine the factor loadings and construct validity (the factors that have values greater than 1 are extracted according to Kaiser-Guttman criterion), and confirmatory factor analysis (CFA) was performed to determine the construct validity of the four-factor DSM-5 model (intrusion, avoidance, negative alterations in cognitions and mood, and hyperarousal), the six-factor anhedonia model (Intrusion, avoidance, negative affect, anhedonia, dysphoric arousal, anxious arousal) [41], and the seven-factor hybrid model of PTSD (Intrusion, avoidance, negative affect, anhedonia, externalizing behaviors, anxiety arousal, dysphoric arousal) [44]. The EFA analysis was conducted using principal component analysis (PCA), with varimax axis rotation and Kaiser normalization. The factor loading of each item in EFA is regarded as acceptable if > 0.5. By CFA, the chi-square, df, chi-square/df, *p*, goodness of fit index (GFI), CFI (comparative fit index), TLI (Tucker-Lewis index), AIC (Akaike information criterion), RMSEA (mean square error of approximation), and SRMR (standardized root mean square residual) were evaluated. Typically, GFI > 0.8 [45], RMSEA < 0.1 and SRMR < 0.08 [46] indicate acceptable model fit, whereas CFI > 0.9 and TLI > 0.9 indicate adequate model fit [47]. Convergent validity was evaluated with Spearman correlations between scores of PCL-5 and CAPS-5, and discriminant validity was tested with Spearman correlations between scores of PCL-5 and questionnaires of anxiety (HADS-A), depression (HADS-D), and alcohol abuse (AUDIT). The diagnostic utility of PCL-5 was calculated with Receiver Operating Curve (ROC) analysis. Specifically, Positive Predictive Power (PPP), Negative Predictive Power (NPP), Overall Correct Classification (OCC), κ (0.5) were reported. Kappa statistics were considered fair agreement for 0.21–0.40, moderate for 0.41 to 0.60, substantial for 0.61 to 0.80, and almost perfect for 0.81–1.0 [48]. The highest value of sensitivity plus specificity was used to determine the optimal cut-off score.

## Results

Descriptive analyses for PCL-5 items indicated positive skewness and leptokurtic (Table 2). The item-total correlation contained no negative values, indicating that the items were assessing the same construct. The descriptive characteristics and internal consistency of PCL-5 and other measures were shown in Table 3. The probable prevalence of anxiety (HADS-A score ≥ 8), depression (HADS-D score ≥ 8) and problem alcohol use (AUDIT score ≥ 8) after stroke attack were 18.97%, 12.93% and 8.91%, respectively. Fourteen participants (4.02%) were formally diagnosed with PTSD through interview. In our stroke patient sample, the overall severity of PTSD symptoms was low and the data were positively skewed. The total scores and all four factors showed good internal consistency (all Cronbach’s α > 0.8 and Guttman’s split-half > 0.7).

**Table 2:** Item-level psychometric properties of the PCL-5

PCL-5 Item	M	SD	Variance	Skew	Kurtosis	Range	Item-total correlation	Cronbach’s α if item deleted
1 (B1)	0.68	1.11	1.23	1.60	1.54	0–4	0.72	0.93
2 (B2)	0.68	1.04	1.08	1.74	2.47	0–4	0.61	0.93
3 (B3)	0.60	0.90	0.82	1.69	2.63	0–4	0.62	0.93
4 (B4)	0.78	0.97	0.93	1.34	1.54	0–4	0.66	0.93
5 (B5)	0.37	0.77	0.59	2.60	7.53	0–4	0.50	0.93
6 (C1)	0.66	1.05	1.10	1.69	2.13	0–4	0.56	0.93
7 (C2)	0.60	1.00	0.99	1.76	2.49	0–4	0.64	0.93
8 (D1)	0.29	0.62	0.38	2.71	9.25	0–4	0.45	0.93
9 (D2)	0.42	0.76	0.58	2.30	5.92	0–4	0.53	0.93
10 (D3)	0.51	0.82	0.68	2.02	4.58	0–4	0.51	0.93
11 (D4)	0.77	0.99	0.99	1.49	2.07	0–4	0.44	0.93
12 (D5)	0.75	1.02	1.04	1.50	1.85	0–4	0.60	0.93
13 (D6)	0.56	0.88	0.77	1.79	3.19	0–4	0.62	0.93
14 (D7)	0.53	0.84	0.71	2.17	5.58	0–4	0.52	0.93
15 (E1)	0.53	073	0.54	1.50	2.66	0–4	0.56	0.93
16 (E2)	0.32	0.65	0.43	2.41	6.62	0–4	0.51	0.93
17 (E3)	0.57	0.78	0.60	1.88	4.90	0–4	0.49	0.93
18 (E4)	0.43	0.77	0.59	2.23	5.69	0–4	0.53	0.93
19 (E5)	0.56	0.82	0.67	2.06	5.35	0–4	0.46	0.93
20 (E6)	0.93	1.09	1.19	1.34	1.30	0–4	0.50	0.93

**Table 3:** Descriptive characteristics and internal consistency of PCL-5 and other measures

	M	SD	Cronbach’s α	Guttman’s split-half	N
Intrusion	3.11	3.90	0.87	0.78	5
Avoidance	1.27	1.88	0.82	0.82	2
Negative alterations in cognition and mood	3.83	4.07	0.81	0.72	7
Alterations in arousal and reactivity	3.35	3.58	0.83	0.82	6
Total PCL-5 score	11.56	11.90	0.94	0.91	20
CAPS-5 score	8.92	10.11	0.92	0.91	20
HADS-A score	4.87	3.82	0.79	0.78	7
HADS-D score	3.99	3.12	0.77	0.77	7
AUDIT	2.54	3.21	0.79	084	10

After checked for audibility and completeness of the 20 audio-recorded CAPS-5 interviews, 19 appeared to be appropriate for the second rating. The inter-rater reliability was high for total PTSD symptom severity score (ICC = 0.97, 95% CI = 0.92–0.99), and was perfect for PTSD diagnosis (κ = 1.0) with agreement on all classifications between raters.

We performed the EFA to see the factor loadings of each item and whether the theoretically expected four factors can be observed in our sample. This sample was suitable for EFA analysis (Kaiser Meyer Olkin = 0.95, chi-square = 3531.97, df = 190, *p* < 0.001). Results showed that the four new factors explained 62.02% of total variance indicating a good representation of the whole data. The factor loadings were presented in Table 4. The analysis demonstrated four factors, although slightly different from the theoretical factor structure. The one to four factors explained 20.68%, 15.71%, 12.96%, and 12.67% of the total variance, respectively. The first factor primarily represented intrusion (4 items) plus avoidance factor (2 items). The second factor primarily represented the factor of alterations in arousal and reactivity (6 items). The factor of negative alterations in cognitions and mood was basically divided into the third (3 items) and fourth factors (3 items). The factor loadings of most items were good (factor loading > 0.5). After removing the 3 items with factor loadings < 0.5 (loss of interest, hypervigilance, and concentration), the factors can explain 65.04% of total variance, slightly elevated compared to the original 20 items.

**Table 4:** Factor loadings of PCL-5 items

PCL-5 Item	Item Description	Factors			
		1	2	3	4
1(B1)	Memories	**0.707**	0.187	0.449	0.219
2(B2)	Dreams	**0.794**	0.212	0.181	0.128
3(B3)	Flashbacks	**0.699**	0.082	0.305	0.225
4(B4)	Cued distress	0.451	0.172	**0.617**	0.189
5(B5)	Cued physical reactions	**0.689**	0.248	0.160	0.168
6(C1)	Avoiding memories	**0.624**	0.377	0.170	0.281
7(C2)	Avoiding external reminders	**0.639**	0.335	0.128	0.314
8(D1)	Dissociative amnesia	0.120	**0.523**	0.309	0.246
9(D2)	Negative beliefs	0.217	0.289	0.162	**0.678**
10(D3)	Blame	0.153	0.177	0.152	**0.770**
11(D4)	Negative feelings	0.239	0.118	0.111	**0.724**
12(D5)	Loss of interest	0.373	0.212	**0.426**	0.396
13(D6)	Detachment/estrangement	0.263	0.297	**0.703**	0.116
14(D7)	Numbing	0.172	0.158	**0.838**	0.135
15(E1)	Irritability or aggressive behavior	0.137	**0.773**	0.135	0.109
16(E2)	Reckless behavior	0.145	**0.614**	0.409	0.269
17(E3)	Hypervigilance	0.420	**0.465**	0.232	0.176
18(E4)	Exaggerated startle	0.448	**0.658**	0.105	0.057
19(E5)	Concentration	0.387	**0.444**	0.139	0.356
20(E6)	Sleep	0.312	**0.572**	0.092	0.326

**Fig. 1:**
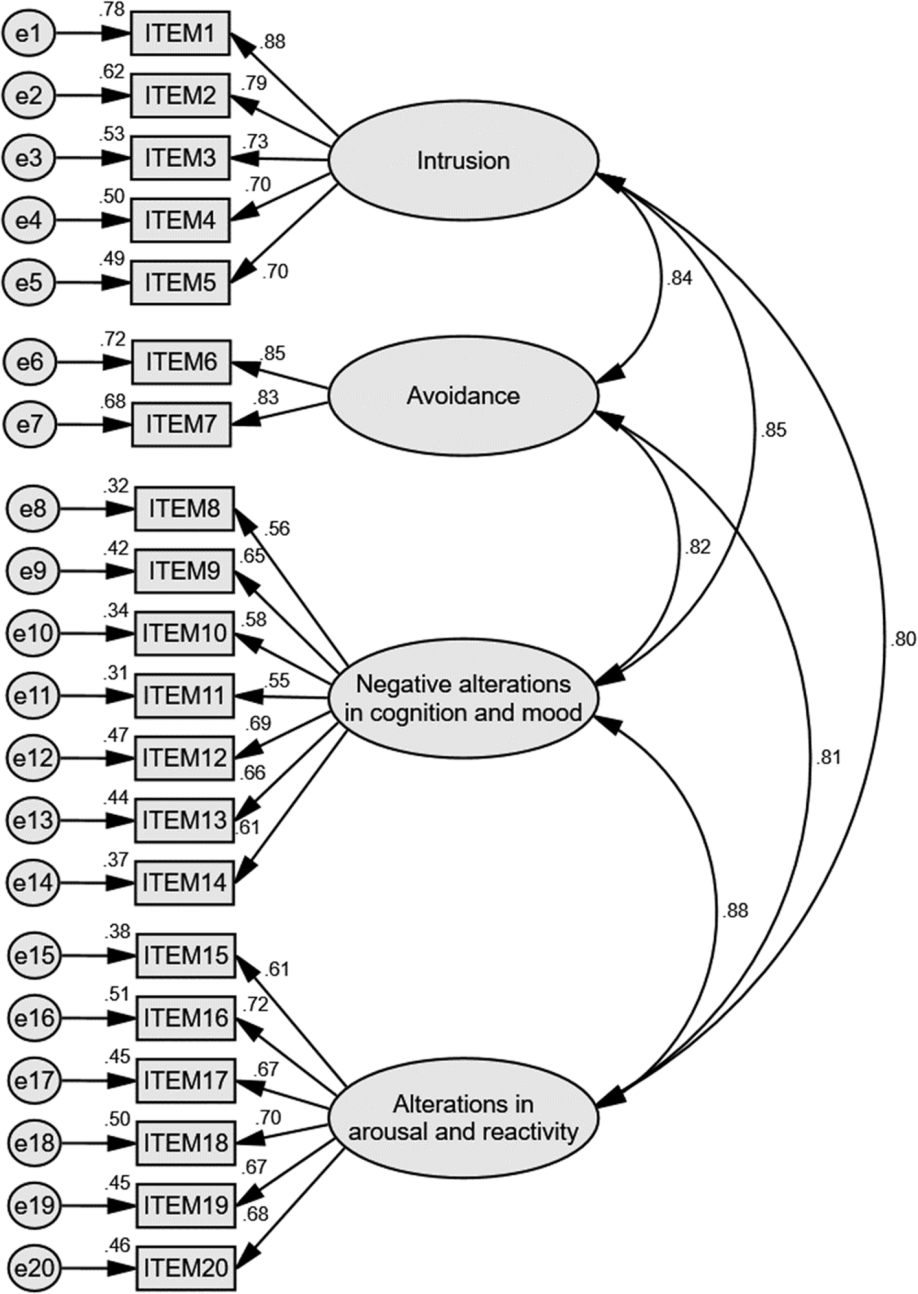
Standardized estimation in CFA analysis results of PCL-5 of the four-factor DSM-5 model. Note. GFI, goodness of fit index; TLI, TuckerLewis index-in comparison with the null model; AIC, Akaike information criterion-in comparison with the null model; CFI, comparative fix index; RMSEA, root mean square error of approximation

**Fig. 2:**
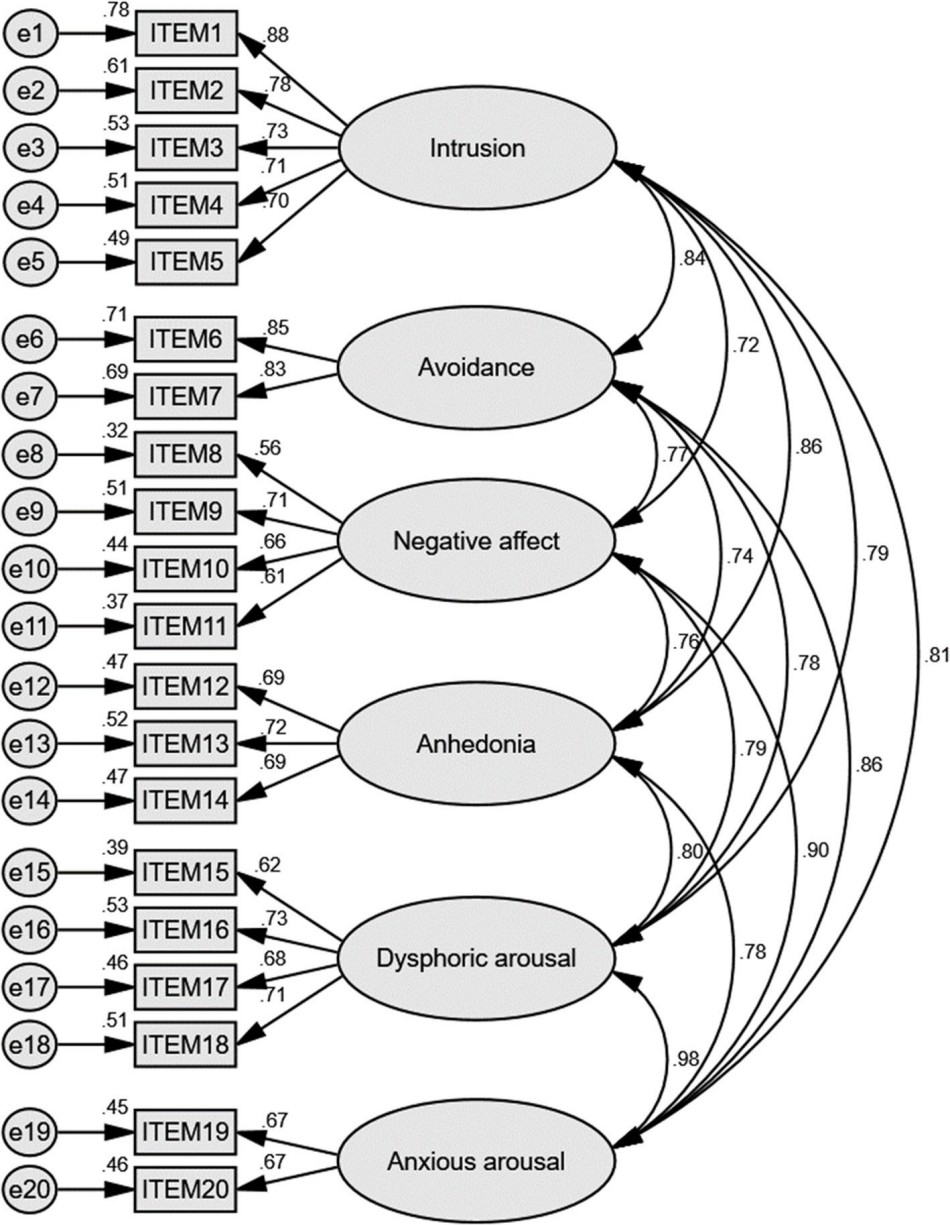
Standardized estimation in CFA analysis results of PCL-5 of the six-factor anhedonia model. Note. GFI, goodness of fit index; TLI, TuckerLewis index-in comparison with the null model; AIC, Akaike information criterion-in comparison with the null model; CFI, comparative fix index; RMSEA, root mean square error of approximation

**Fig. 3:**
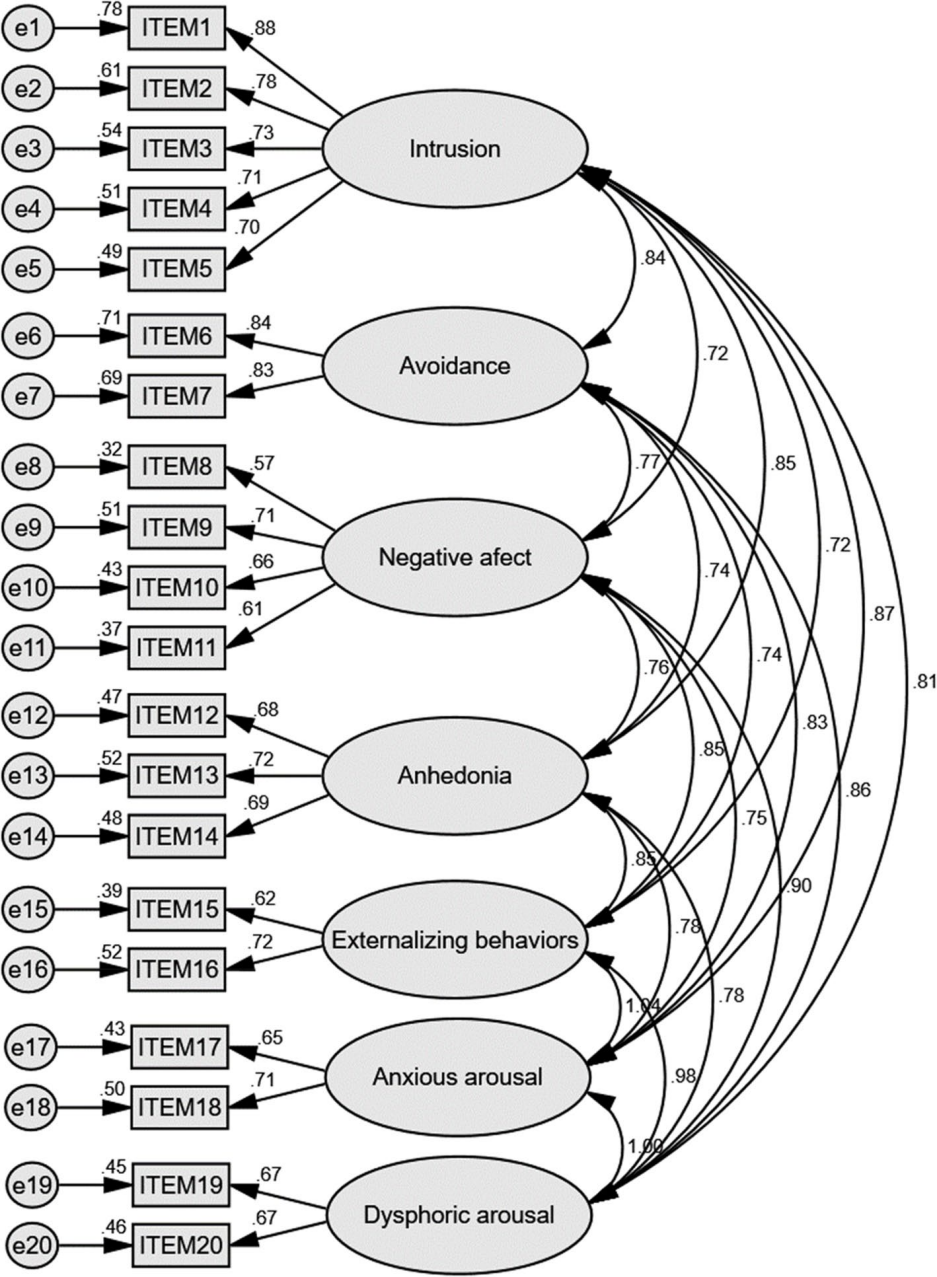
Standardized estimation in CFA analysis results of PCL-5 of the seven-factor hybrid model. Note. GFI, goodness of fit index; TLI, TuckerLewis index-in comparison with the null model; AIC, Akaike information criterion-in comparison with the null model; CFI, comparative fix index; RMSEA, root mean square error of approximation

CFA were then used to evaluate the PCL-5 with original four factors (Fig. 1; Table 5). Factor loadings of all item were acceptable (ranged from 0.55 to 0.88). Parameters such as chi-square, GFI (> 0.8), CFI (> 0,9), TLI (> 0.9), AIC, RMESA (< 0.08), standardized RMR (= 0.048) indicated good model fit [45–47, 49]. The six-factor anhedonia model and the seven-factor hybrid model of PTSD were also assessed by CFA and both showed better model fit than the original four-factor model, with the hybrid model demonstrated the best (Figs. 2 and 3; Table 5).

**Table 5:** Confirmatory factor analysis of different models of PCL-5

	DSM-5 model	Anhedonia model	Hybrid model
Chi-square (χ^2^)	428.238	364.026	333.800
df	164	155	149
Chi-square/df	2.611	2.349	2.240
*p*	< 0.001	< 0.001	< 0.001
Goodness of Fit Index (GFI)	0.886	0.904	0.911
Comparative Fit Index (CFI)	0.923	0.939	0.946
Tucker-Lewis Index (TLI)	0.910	0.925	0.931
Akaike Information Criterion (AIC)	520.238	474.026	455.800
Mean Square Error of Approximation (RMSEA)	0.068	0.062	0.060
Standardized Root Mean Square Residual (SRMR)	0.0478	0.0454	0.0433

By Spearman correlation test, PCL-5 total scores and subscale scores all showed moderate to strong positive correlations with PTSD criteria in CAPS-5 (Table 6). This implied good convergent validity of PCL-5. PCL-5 total score also showed strong positive correlations with total scores of HADS-A and HADS-D for the entire sample, however, it did not demonstrate correlations with AUDIT (Table 7).

**Table 6:** Correlations among PCL-5 factors and CAPS-5 criterions (Bivariate Spearman rho)

	Intrusion	Avoidance	Negative alterations in cognitions and mood	Alterations in arousal and reactivity	PCL-5 total score
CAPS-5 B	.742^**^	.549^**^	.546^**^	.483^**^	.711^**^
CAPS-5 C	.579^**^	.747^**^	.487^**^	.418^**^	.632^**^
CAPS-5 D	.520^**^	.449^**^	.633^**^	.453^**^	.600^**^
CAPS-5 E	.495^**^	.466^**^	.439^**^	.568^**^	.539^**^

^**^P < 0.01 (2-tailed)

**Table 7:** Discriminant validity correlations for the PCL-5 and other measures

Measure	1	2	3	4	5
1. PCL-5	1.00				
2. CAPS-5	0.85^**^	1.00			
3. HADS-A	0.73^**^	0.68^**^	1.00		
4. HADS-D	0.74^**^	0.66^**^	0.58^**^	1.00	
5. AUDIT	0.07	0.07	0.09	0.04	1.00

^**^P < 0.01 (2-tailed)

By ROC analysis, the PCL-5 total score’s area under the curve (AUC) was 0.99 (SE = 0.003) indicating a very good level of diagnostic accuracy. The optimal cutoff scores of PCL-5 for the PTSD diagnosis as assessed through the CAPS-5 in our sample was 37, which had the highest sensitivity and specificity (Table 8).

**Table 8:** Various PCL-5 cutoff scores in predicting PTSD diagnosis with DSM-5 standard

Cutoff score	Sensitivity	Specificity	PPP	NPP	OCC	κ (0.5)
31.5	1.000	.949	0.438	1	0.948	0.585
32.5	1.000	.955	0.483	1	0.957	0.631
33.5	1.000	.970	0.583	1	0.971	0.723
34.5	1.000	.973	0.609	1	0.974	0.744
35.5	1.000	.976	0.636	1	0.977	0.766
37.0	1.000	.979	0.667	1	0.980	0.790
38.5	.929	.982	0.684	0.997	0.980	0.778
40.5	.929	.985	0.722	0.997	0.983	0.804
42.5	.929	.988	0.765	0.997	0.986	0.831
43.5	.857	.991	0.80	0.994	0.986	0.820
44.5	.714	.991	0.769	0.988	0.980	0.730
45.5	.714	.994	0.833	0.988	0.983	0.760

PPP (Positive Predictive Power) = true positives/ (true positives + false positives); NPP (Negative Predictive Power) = true negatives/ (true negatives + false negatives); OCC (Overall Correct Classification) = (true positives + true negatives)/ (true positives + true negatives + false positives + false negatives); κ (0.5) = quality of efficiency

## Discussion

Although many scales have been designed for assessing PTSD symptoms, there lacks a reliable and valid screening instrument specifically designed for post-stroke PTSD, which has been increasingly drawing interest. Our study was the first to evaluate the psychometric properties of the PCL-5 among the Chinese stroke patients. Our results indicated the PCL-5 has a good factor structure in assessing the current sample. Additionally, we found that the overall PTSD symptoms in our sample was relatively low with a 4% of diagnosed PTSD rate. A cutoff score of 37 for diagnostic value of PCL-5 was suggested by our study.

According to previous studies, prevalence or incidence of post-stroke PTSD varied greatly (from 4 to 37%) [14]. The PTSD rate and symptom severity in our sample were relatively low. This may be due to the different social and cultural background of the Chinese population. Moreover, participants in our sample were all outpatients, potentially excluding those with severe psychiatric or neurologic symptoms. Also, the majority of participants were male (73%), who may be less prone to develop PTSD [25, 50]. The selection bias may lead to milder PTSD symptoms in our study. Additionally, despite heterogeneity in methodology, previous small sample studies indicated the PTSD symptoms after stroke gradually decreased with the times. A meta-analysis in revealed the estimated rate of PTSD/PTSS following stroke or TIA was 23% within 1 year and 11% after 1 year [51]. More than half of our participants had stroke courses longer than 1 year. Future studies with large sample size and longitudinal design are required to further elucidate the temporal variation of PTSD rates after stroke and the possibility of delayed-onset PTSD.

In previous studies of other populations, the Cronbach’s alpha value of PCL-5 total score ranged from 0.91 to 0.97, with that of individual item ranged from 0.79 to 0.93 [4–6]. The Cronbach’ alpha in our sample was also excellent (total score, 0.94; individual item, 0.81 to 0.87).

Since models of EFA and CFA are different, previous studies showed that factor structures obtained by EFA often turn out to fit poorly in CFA [4, 52]. Thus we conducted both analyses. In the EFA, four factor model was detected, but was different from the theoretical model. The most prominent structure discrepancies were that the factor of avoidance was integrated into the factor of intrusion, and the factor of negative alterations in cognitions and mood was split into two factors. This result was quite similar to the previous study on Greek women after cesarean section [4]. The possibility of symptom overlaps or interdependence of the two symptom clusters of intrusion and avoidance has been proposed by some previous studies in different samples [6, 53]. In a study of Chinese adolescents surviving an earthquake, analyses similarly split the PCL-5 factor of negative alterations in cognitions and mood into negative affect factor and anhedonia factor [54]. Our results were consistent with this study. In our sample, after deleting the factors with loadings < 0.5, the total variance explained only increased slightly (by 3.02%), thus the factor’s structure was reserved.

According to EFA, loss of interest (“Loss of interest in activities that you used to enjoy?”), hypervigilance (“Being “superalert” or watchful or on guard?”) and concentration (“Having difficulty concentrating?”) are items with factor loadings of < 0.5. This may be explained by the specificity of stroke. Stroke often causes neurologic deficits such as limb weakness, bradylalia, and dizziness, which may very well lead to loss of some interests (e.g., sports, drinking, and socializing); Stroke has the potential of recurrence and exaggeration, and it may be effectively treated with thrombolysis or thrombectomy if detected in the super early stage after attack. Thus, patients are generally taught to be alert on the initial evidence of recurrence; Additionally, stroke patients are commonly individuals with older ages (averaging 55.41 years old in our sample) who have reduced energy compared to younger ones, and the lesion in brain may more or less influence cognition and concentration. These above symptoms are the results of stroke itself and may not represent PTSD symptoms.

Through CFA analysis, the PCL-5 also conformed to the original dimensions, meaning the four-factor structure worked adequately for the Chinese stroke patients. Besides the DSM-5 model, previous studies also proposed other models such as Anhedonia model [41] and hybrid model [44] for PTSD. In our study, the seven-factor hybrid model exhibited the best fit. This result was in accordance with the findings on diverse populations [6, 40, 55–60]. Thus, whether the DSM-5 model remains the best fit in specific populations is left for future research. In our sample, the PCL-5 scores demonstrated good convergent validity with CAPS-5 criteria, with correlations ranging from 0.568 to 0.747. Additionally, the PCL-5 scores also showed good discriminant validity by being strongly correlated with scales evaluating anxiety (HADS-A) and depression (HADS-D) while being not correlated with alcohol habits (AUDIT). These results were consistent with previous studies, except for the weak correlation between PCL-5 and AUDIT [5, 6]. This may be due to the reason that stroke patients generally quit drinking as advised by doctors and most of the participants in our sample only had mild or no PTSD symptoms, not severe enough to influence drinking habits.

For the Chinese version of the PCL-5, a cutoff point of 37 was the one that had the best predictive values (Youden index J = 0.979). According to previous studies, the cutoff scores of PCL-5 was suggested ranging from 25 to 45 [5, 6, 61–64] in different populations. Our indicated value of 37 was average compared to these studies. Since mental disorders may negatively affect stroke patients’ prognosis, it is critical to screen PTSD in this population. However, due to the large number of stroke patients, there lacks enough neurological psychologists/psychiatrists for PTSD interview. Our study suggested that the self-report PCL-5 can fulfill the need of initial screening. In this sample a cut-off of 37 provided a sensitivity of 1.00 and a specificity of 0.979, demonstrating a high degree of diagnostic accuracy. It should be pointed out that whether and at what level to set a cut-off and caseness scores is based on the purpose for using the scale [65]. It is also of important clinical value to detect individuals reporting significant subthreshold symptoms, since there are instances where treatment may benefit these individuals who are experiencing clinically significant functional impairment [6]. With the popularization and application of PCL-5 for initial screening and follow-up assessment, PTSD symptoms can be easily monitored facilitating future studies on the early intervention for post-stroke PTSD.

There were some limitations of our study. First, in our sample, the proportion of formally diagnosed PTSD was small and the overall PTSD symptoms was low. This may influence the analysis of cutoff value. Studies on samples with higher rate of PTSD patients are required in the future. Second, since social and cultural background can influence the psychometric properties, our results may not necessarily be translated to stroke populations in other races or societies. Third, because previous studies showed that PCL-5 is correlated with alcohol abuse [66, 67], we initially included AUDIT. However, the use of the AUDIT scale has not been validated in stroke patients, thus its application as a criterion for convergent and divergent validity may be debated. Fourth, in the study, we only aimed to investigate the psychometric properties of measures associated with PTSD, so we did not collect information on history of psychiatric disorders, which may be an important exclusion criterion in studies on epidemiological studies on post-stroke PTSD, revealing its prevalence and risk factors.

## Conclusion

Our findings support use of the PCL-5 as a psychometrically adequate measure of post-stroke PTSD in the Chinese patients.

## Data Availability

The datasets used and/or analyzed during the current study are available from the corresponding author [HB] on reasonable request.

## Abbreviations

PTSD: Post-traumatic stress disorder
PCL-5: Post-traumatic Stress Disorder (PTSD) Checklist for DSM-5
CAPS-5: Clinician Administered PTSD Scale for DSM-5
ROC: Receiver operating characteristic
TIA: Transient ischemic attack
SAH: Subarachnoid hemorrhage
EFA: Exploratory factor analysis
CFA: Confirmatory factor analysis
